# Puerarin Attenuates Ovalbumin-Induced Lung Inflammation and Hemostatic Unbalance in Rat Asthma Model

**DOI:** 10.1155/2014/726740

**Published:** 2014-01-02

**Authors:** Feng Dong, Chengbin Wang, Jinyan Duan, Weiyi Zhang, Daijun Xiang, Mianyang Li

**Affiliations:** Department of Clinical Laboratory Medicine, General Hospital of Chinese People's Liberation Army, Beijing 100853, China

## Abstract

*Aim.* We aimed to investigate and evaluate the preventive activity of puerarin on the ovalbumin-induced asthma rat model.* Materials and Methods. *Male Wistar rats were sensitized intraperitoneally on days 0, 7, and 14 and challenged to ovalbumin intratracheally on day 21. Groups of sensitized rats were treated randomly either with placebo, puerarin, dexamethasone, or puerarin combined with dexamethasone, from days 15 to 20. Inflammatory markers, including cell counts in bronchoalveolar lavage fluid (BALF), inflammatory cytokines, histopathology, and coagulation parameters, such as coagulation tests and the activity of coagulation factors, were analyzed.* Results. *Puerarin significantly inhibited the recruitment of inflammatory cells in BALF and lung tissue. At the same time, the release of IL-4, IL-10, and IFN-**γ** in serum and the expression of mRNAs in lung tissue homogenate were changed by puerarin. Administration of puerarin also effectively rectified the coagulation disorder in asthmatic rats, such as prothrombin time (PT) (*P* < 0.01), thrombin time (TT) (*P* < 0.05), fibrinogen (FIB) (*P* < 0.01),the activity of factor II (FII) (*P* < 0.01), the activity of factor V (FV) (*P* < 0.05), the activity of factor VII (FVII) (*P* < 0.05), the activity of factor X (FX) (*P* < 0.05), the activity of factor VIII (FVIII) (*P* < 0.01), the activity of factor IX (FIX) (*P* < 0.05), and the activity of factor XII (FXII) (*P* < 0.05).* Conclusions. *Our results provide a clue that puerarin was useful for the preventive of allergic airway disease in rodents.

## 1. Introduction

Asthma is a chronic inflammatory disease of the airways characterised by eosinophilic infiltration, airway hyperresponsiveness, and structural remodeling [1–3], along with hemostatic unbalance [4, 5]. Asthma morbidity and mortality have been increasing in recent decades [6–8]. Currently, there is no direct effective therapy for asthma, only symptomatic treatment. Asthma cannot be completely healed or cured and thus needs continuous medical treatment [9].

At present, inhaled corticosteroids and *β*-2-agonists are used as the first line of treatment of asthma for reducing airway inflammation and bronchial constriction [10, 11]. Furthermore, relapse after therapy withdrawal is common [12], and the effects of these drugs are not always satisfactory in clinical practice because of local or systemic side effects [13–15]. On the other hand, although corticosteroids improve asthma symptoms, they do not alter the progression of asthma or cure the disease [16]. Thus, new or alternative approaches are being tried to the control of asthma.

Puerarin(8-(beta-D-Glucopyranosyl-7-hydroxy-3-(4-hydroxyphenyl)-4H-1-benzopyran-4-one) [17], with molecular formula C21H20O9, is extracted from the herbal medicine Radix Puerariae. Previous studies revealed that puerarin might possess many physiological and pharmacological activities and has long been used for cardiovascular diseases, such as coronary artery disease, myocardial damage, and heart failure [18–20]. Puerarin also shows antioxidative [21] and antiallergic activities [22]. Particularly, for asthma, disturbed hemostatic balance is one of the important factors in asthma for the perpetuation of allergic inflammation [4]. In our early research, we found that puerarin could inhibit the release of inflammatory cytokines in the coculture of human bronchial epithelial cells and neutrophils model [20, 23]; furthermore, puerarin could effect the cell level of prevention and treatment of bronchial asthma [24]. In this study, we used the ovalbumin- (OVA-) induced asthmatic rat model to evaluate the preventive effect of puerarin to make a further research.

## 2. Materials and Methods

### 2.1. Chemicals and Reagents

Puerarin (purity 99.4%) was purchased from Han Yin HEYEMAIDISEN Plant Pharmaceutical Co., Ltd., China. Dexamethasone sodium phosphate injection was obtained from Tanjin Pharmaceutical Co., Ltd., China. Ovalbumin was purchased from Sigma-Aldrich Inc., USA. Alum was purchased from Thermo Pierce Inc., USA. The other chemicals and reagents used were of analytical grade.

### 2.2. Animals

50 male Wistar rats (SPF grade, 4 weeks old) were supplied by the laboratory animal center of the PLA General Hospital, Beijing, China. Filtered tap water and commercial diet were available ad libitum. Rats were housed in pathogen-free cages kept at the temperature of 20–25°C and 50–70% relative humidity. This study was conducted in accordance with the internationally accepted principles for laboratory animal use and care according to the US guidelines (NIH Publication no. 85-23, revised in 1985), and the study was approved by the Ethics Committee of the Medical College of the People's Liberation Army (PLA).

### 2.3. Induction of Asthma and Drug Administration

The rats were randomly divided into five groups (10 rats in each). The normal group was sensitised, challenged, and treated with normal saline (NS, NaCl 0.9%). The experimental groups were sensitized intraperitoneally (I.P.) with 1 mg OVA adsorbed on 20 mg Al(OH)_3_ gelatinous on days 0, 7, and 14. They were challenged with 1.1% OVA in 200 *μ*L normal saline by intratracheal instillation on day 21. From 15 d to 20 d, OVA sensitized and challenged rats were treated with either (1) the asthma group: 1 mL NS (orally) and 1 mL NS (I.P); (2) the pue (puerarin) group: 100 mg/kg puerarin (orally) and 1 mL NS (I.P); (3) the pue+dex (dexamethasone) group: 100 mg/kg puerarin (orally) and 0.5 mg/kg dexamethasone (I.P); (4) the dex group: 1 mL NS (orally) and 0.5 mg/kg dexamethasone (I.P). The rats were sacrificed on day 22.

### 2.4. Blood Collection

24 hours after the challenge, rats were anesthetized by intraperitoneal injection of 4% chloral hydrate (40 mg/kg). Then, the animals were dissected. And then the animals were euthanized by aortic exsanguination. The artery blood was collected in different tubes with sodium citrate anticoagulant or gel procoagulant for different tests.

### 2.5. Inflammatory Cell Counts in Bronchoalveolar Lavage Fluid (BALF)

After euthanization and opening of chest cavity, rats lungs were thoroughly examined for the morphological lesions. Then, a cannula was inserted into the trachea *in situ* and the left lung was lavaged with 2.5 mL normal saline. The fluid was collected by gentle aspiration and this procedure was repeated 3 times. The cell pellet in BALF was spin down and resuspended in 1 mL of NS. Leukocyte numbers were determined in a Neubauer counting chamber. The eosinophil cell count was assessed on slides prepared by a cytospin centrifuge (Xiangyi, China). After Wright's staining, eosinophils were identified under the light microscope at 1000x magnification. At least 400 cells were differentiated on each slide.

### 2.6. Lung Tissues Histopathology

After BAL processing, the right lungs were fixed in 10% neutral buffered formalin. Each tissue was sliced into 4 *μ*m sections and stained with H&E stain (Mayer's hematoxylin & eosin). Tissue lesions and inflammatory cell infiltration were then examined using microscope.

### 2.7. IL-4, IL-10, and IFN-*γ* Measurements

The level of IL-4, IL-10, and IFN-*γ* in serum was assessed according to the manufacturers' instructions using rat IL-4, IL-10, IFN-*γ* ELISA kit (R & D Systems, USA).

### 2.8. Blood Coagulation Test of Prothrombin Time (PT), Activated Partial Thromboplastin Time (APTT), Thrombin Time (TT), and Fibrinogen (FIB)

2.7 mL rat blood was collected in vacuum tube with sodium citrate. The platelet deficiency plasma was obtained by centrifugation (3000 rpm, for 10 min); then blood coagulation test was conducted with STA-R evolution automatic blood coagulation test instrument (Stago Inc., France) according to the manufacturer's instructions within 2 hours.

### 2.9. Activity of Coagulation Factor

2.7 mL rat blood was collected in vacuum tube with sodium citrate. The platelet deficiency plasma was obtained by centrifugation (3000 rpm, for 10 min); then the activity of coagulation factor was detected by ACL-Top 700 hemostasis analyzer (IL Inc., USA) according to the manufacturer's instructions within 2 hours.

### 2.10. RNA Preparation and Quantitative RT-PCR

The total RNA from lung tissues was isolated with the use of TRIzol (Solarbio, Beijing, China). Real-time PCR was performed on cDNA samples using the SYBR Green system (Tian Long Technology Co., Ltd., China). Primers are designed as Table 1. Cycling conditions were as follows: 96°C for 180 seconds, followed by 35 cycles of 94°C for 30 seconds, 47°C for 30 seconds, 72°C for 60 seconds, 95°C for 60 seconds, and 53°C for 60 seconds. Analysis was performed using the sequence detection software supplied with the instrument. The relative quantitation value is expressed as 2 ± DCT, where DCT is the difference between the mean CT value of duplicates of the sample and of the GADPH control.

### 2.11. Statistical Analysis

All data were expressed as means ± standard errors of the mean (SEMs). All analysis was performed using the statistical package for the social sciences (SPSS) statistical software for Windows, version 17.0 (SPSS Inc., USA). The statistical significance of differences was assessed by one-way ANOVA. *P* < 0.05 was considered to be significantly different. When ANOVA indicated a significant difference, LSD or Dunnett's T3 post hoc test was then used to assess the difference between groups.

## 3. Results

### 3.1. Effect of Puerarin on OVA-Induced Eosinophilia in BALF

**Figure 1:**
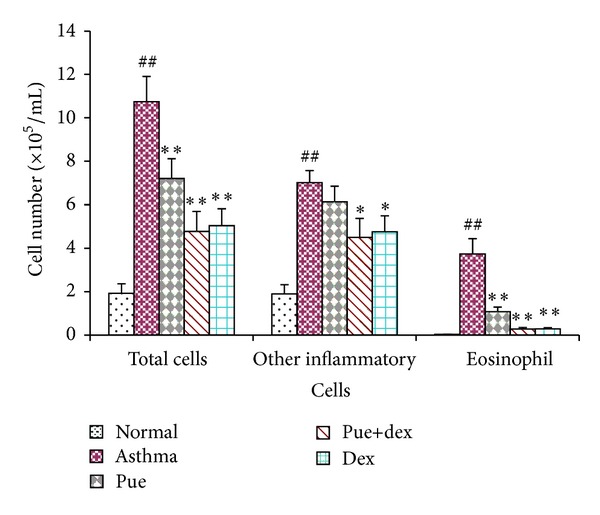
Effect of puerarin on recruitment of inflammatory cells in BALF of rat after the last OVA challenge. Values are expressed as means ± SEMs (*n* = 10/group). ^#^ *P* < 0.05 and ^##^ *P* < 0.01 when compared with normal control group; *P < 0.05 and **P < 0.01 when compared with asthma group.

As shown in Figure 1, compared with normal rats, OVA caused a marked leukocyte influx into BALF in the asthma group (1.92 ± 0.43  × 10^5^/mL versus 10.74 ± 1.16  × 10^5^/mL, *P* < 0.01). Eosinophils constituted less than 1.5% of total leukocytes in normal rats. However, eosinophil levels dramatically increased up to more than 30% of total leukocytes, in the BALF of asthma rats. In rats treated with puerarin, cell migration was significantly attenuated, and a significant decrease in total leukocytes (10.74 ± 1.16  × 10^5^/mL versus 7.21 ± 0.91  × 10^5^/mL, *P* < 0.01) and an obvious drop in eosinophils (31.97 ± 4.04% versus 13.76 ± 1.23%, *P* < 0.01) were observed, compared to the asthma group. A positive drug control, dexamethasone, showed a similar suppressive effect on leukocyte influx into BALF.

### 3.2. Effect of Puerarin on OVA-Induced Eosinophilia in Lung Tissue

**Figure 2:**
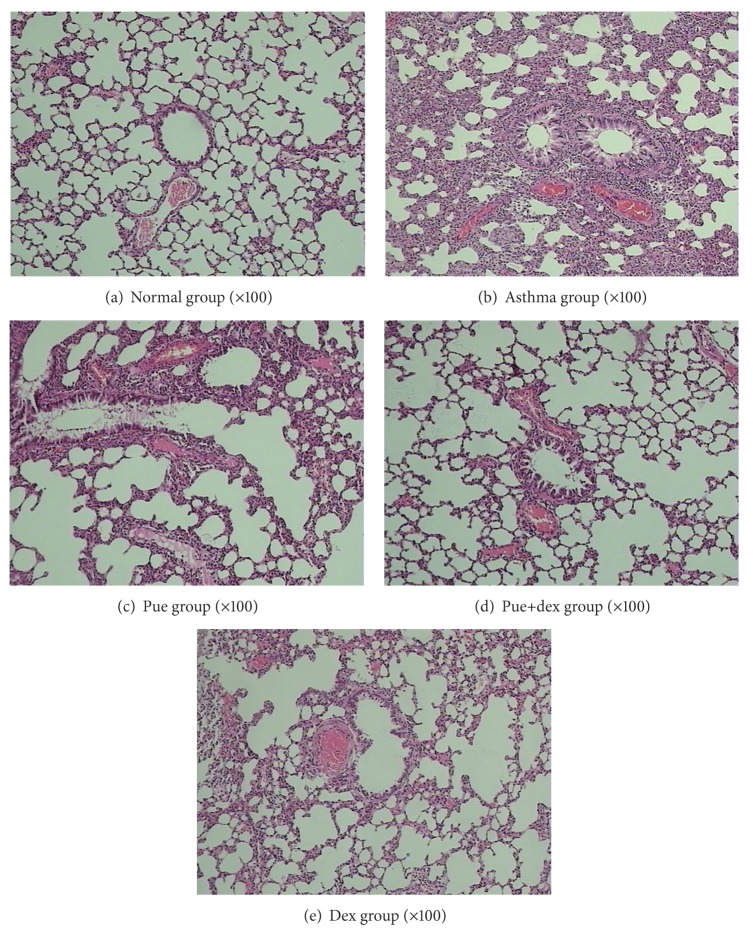
Effect of puerarin on recruitment of leukocytes in lung tissue. Lung tissues were fixed, sectioned at 4 *μ*m thickness, and stained with H&E solution.

Compared with the normal control group, numerous eosinophils were observed in the lung interstitium around airways and blood vessels, along with narrowing of the airway lumen in the asthma group (Figures 2(a) and 2(b)). In drugs prevention groups, all of them significantly reduced the inflammatory cell infiltration and mucus production (Figures 2(c)–2(e)), compared with the asthma group. Puerarin treatment significantly downregulated the accumulation of mucus in the airways and prevented the accumulation of eosinophils in BALF after OVA challenge (Figures 2(b) and 2(c)). The rats treated by dexamethasone or dexamethasone combined with puerarin showed a substantial reduction in inflammation and eosinophil infiltration into lungs (Figures 2(d) and 2(e)).

### 3.3. IL-4, IL-10, and IFN-*γ* Measurement

**Figure 3:**
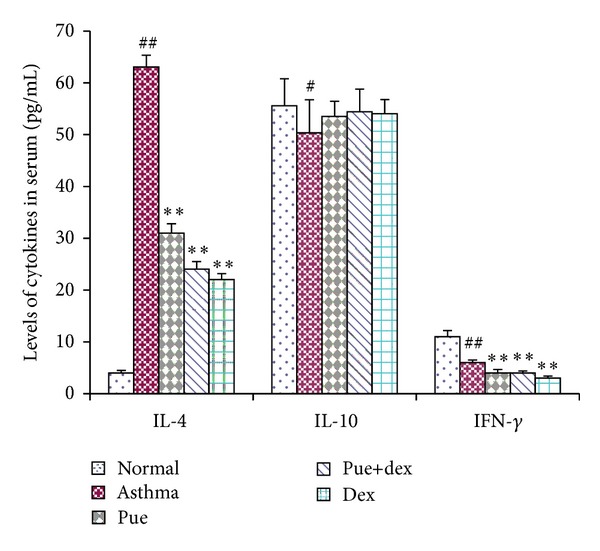
Effect of puerarin on serum IL-4, IL-10, and IFN-*γ*. Values are expressed as means ± SEMs (*n* = 10/group). ^#^ *P* < 0.05 and ^##^ *P* < 0.01 when compared with normal control group; *P < 0.05 and **P < 0.01 when compared with asthma group.

Significant changes of IL-4, IL-10, and IFN-*γ* were observed in the asthma group in comparison to the normal group (*P* < 0.01, *P* < 0.05, and *P* < 0.01). Moreover, the IL-4 and IFN-*γ* levels of puerarin treatment group were obviously lower than the asthma group, as shown in Figure 3.

### 3.4. Effect of Puerarin on PT, APTT, TT, and FIB

**Figure 4:**
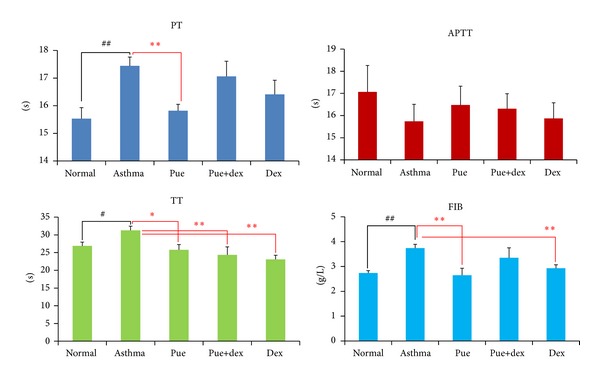
Effects of puerarin on PT, APTT, TT, and FIB. Values are expressed as means ± SEMs (*n* = 10/group). ^#^ *P* < 0.05 and ^##^ *P* < 0.01 when compared with normal control group; *P < 0.05 and **P < 0.01 when compared with asthma group.

PT, TT, APTT, and FIB assays were used to evaluate the effect of puerarin on blood coagulation. As shown in Figure 4, compared with the asthma group, the normal group had significantly lower PT (*P* < 0.01), FIB (*P* < 0.01), and TT (*P* < 0.05); the APTT was higher but insignificantly (*P* = 0.281); puerarin could significantly reduce PT (*P* < 0.01), FIB (*P* < 0.01), and TT (*P* < 0.05); however, puerarin combined with dexamethasone showed less potent effect on such parameters, just obviously reducing TT (*P* < 0.01); and dexamethasone treatment could significantly decrease FIB (*P* < 0.05) and TT (*P* < 0.01).

### 3.5. Effect of Puerarin on the Activity of Coagulation Factor in Extrinsic Pathway

**Figure 5:**
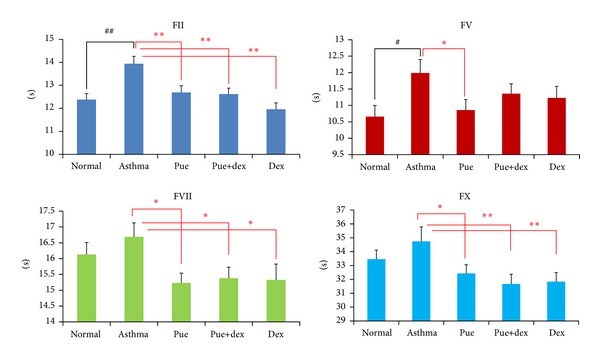
Effect of puerarin on the activity of coagulation factor in extrinsic pathway. Values are expressed as means ± SEMs (*n* = 10/group). ^#^ *P* < 0.05 and ^##^ *P* < 0.01 when compared with normal control group; *P < 0.05 and **P < 0.01 when compared with asthma.

As shown in Figure 5, compared with the asthma group, the normal group was of higher coagulation factors FII and FV activities (*P* < 0.01 and *P* < 0.05, resp.); puerarin could significantly enhance the activities of FII, FV, FVII, and FX (*P* < 0.01, *P* < 0.05, *P* < 0.05, and *P* < 0.05, resp.); puerarin combined with dexamethasone could also enhance the activities of FII, FVI,I and FX (*P* < 0.01, *P* < 0.05, and *P* < 0.01, resp.), just the same as the dexamethasone treatment group.

### 3.6. Effect of Puerarin on the Activity of Coagulation Factor in Intrinsic Pathway

**Figure 6:**
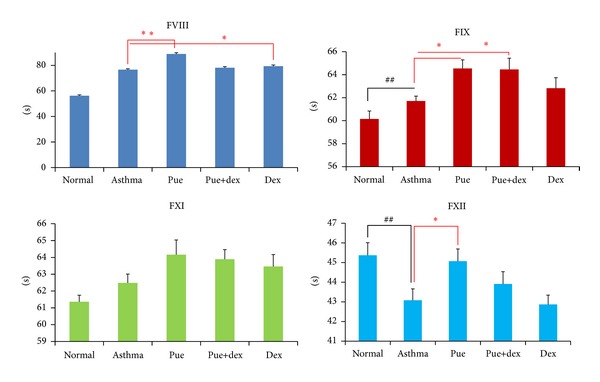
Effect of puerarin on the activity of coagulation factor in intrinsic pathway. Values are expressed as means ± SEMs (*n* = 10/group). ^#^ *P* < 0.05 and ^##^ *P* < 0.01 when compared with normal control group; *P < 0.05 and **P < 0.01 when compared with asthma group.

As shown in Figure 6, compared with the asthma group, the normal group was of higher FVIII activities (*P* < 0.01) and lower FXII activity (*P* < 0.01); puerarin could obviously reduce the activities of FVIII, FIX, and FXII (*P* < 0.01, *P* < 0.05, and *P* < 0.05, resp.); puerarin combined with dexamethasone showed less effectiveness on such parameters, just obviously decreasing the activity of FIX (*P* < 0.01); however, dexamethasone treatment significantly reduced the activity of FVIII (*P* < 0.05).

### 3.7. Effect of Puerarin on mRNA Expression

**Figure 7:**
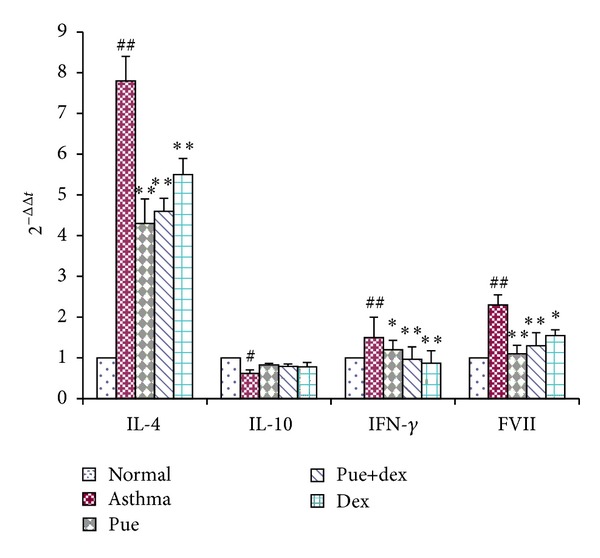
Effect of puerarin on the expression of mRNA in asthmatic rats lung tissue. Values are expressed as means ± SEMs (*n* = 10/group). ^#^ *P* < 0.05 and ^##^ *P* < 0.01 when compared with normal control group; *P < 0.05 and **P < 0.01 when compared with asthma group.

As shown in Figure 7, a high level of anti-inflammatory IL-10 mRNA was expressed in normal rats. However, IL-10 mRNA expression was markedly downregulated in asthmatic rats (*P* < 0.05); yet no significant change was found in different drugs preventive group. On the other hand, asthmatic rats had a significant increase in the level of IL-4 mRNA, IFN-*γ* mRNA, and FVII mRNA over those of normal rats (*P* < 0.01, *P* < 0.01, and *P* < 0.01). Furthermore, puerarin could markedly suppress the IL-4 mRNA, IFN-*γ* mRNA, and FVII mRNA expression in asthmatic rats (*P* < 0.01, *P* < 0.05, and *P* < 0.01). Meanwhile, dexamethasone could also inhibit those mRNA expressions in asthmatic rats (*P* < 0.01, *P* < 0.01, and *P* < 0.05).

## 4. Discussion and Conclusion

Puerarin (4′-7-dihydroxy-8-*β*-D-glucosylisoflavone) is a C-glycoside compound extracted from Gegen. A number of studies have revealed that puerarin possesses many biological activities, including the improvement of blood circulation [25], prevention of cardiovascular diseases [26], antipyretic [27], antioxidative [21] and antiallergic activities [22].

Asthma is a disease of chronic airway inflammation causing symptoms of paroxysmal airflow obstruction, airway hyperresponsiveness to irritative stimuli, wheezing, chest tightness, and coughing [28]. These symptoms occur against a background of allergic inflammation, characterized by the infiltration of mast cells, eosinophils, and lymphocytes into the airway wall and causing mucus hypersecretion [4, 29, 30]. The pulmonary histological assay (Figure 2) evidences that various degrees of inflammation appeared in all OVA-treated cases. However, the asthma rats presented with more severe inflammation (inflammatory cell influx) than the treatment groups, and generally more serious inflammation was found in the puerarin group than the dexamethasone or puerarin combined with dexamethasone group. However, it is worth noting that puerarin-treated rats showed a significant reduction in inflammatory cell infiltration and mucus production compared to asthmatic rats. Additionally, the total cell number in BALF of asthmatic rats was significantly higher than other groups (*P* < 0.01, Figure 1), and rats treated with drugs showed substantial decrease compared with asthmatic rats (*P* < 0.01, Figure 1). One of the basic features of allergic asthma has been shown to be the eosinophilic airway inflammation [1, 31]. Therefore, eosinophilic percentage was examined as a major biomarker for allergic asthma; our results indicated that drugs used in this study could effectively reduce the eosinophil numbers in BALF (*P* < 0.01, Figure 1). Importantly, compared with the asthmatic group, the number of eosinophils was substantially reduced in puerarin-treated rats (*P* < 0.01, Figure 1). Thus, both inflammatory cell counts in BALF and pulmonary histological assay suggested that puerarin could significantly suppress the airway inflammation of asthma. Allergic asthma is characterized by unbalance of Th1/Th2 cell and recruitment of type 2 T helper (Th2) cells [32, 33]. Th1 cells mainly excrete cytokines like IL-2, IFN-*γ*, and TNF-*β*; Th2 cells mainly excrete cytokines IL-4, IL-5, IL-6, IL-9, and IL-13. These two form a cytokine network by the way of self-enhancement and mutual antagonism [34]. We observed that puerarin had an effect on the Th1/Th2 balance. Firstly, compared with normal rats, the concentration of IL-4 in serum was up while the IFN-*γ* was down in asthmatic rats. Secondly, the IL-4 and IFN-*γ* mRNA expression in lung tissue homogenate obviously higher in asthmatic rats than normal rats. Thirdly, the results also show that puerarin can inhibit the serum IL-4 and IFN-*γ* levels in asthmatic rats while increase the IL-10. IL-10 is produced by a subset of Tregs and by macrophages [35]. And IL-10 is a potent anti-inflammatory cytokine that inhibits the synthesis of many inflammatory proteins, including allergic inflammation-related cytokines (like tumor necrosis factor-*α*, granulocyte-macrophage colony-stimulating factor (GM-CSF), IL-5, and several chemokines) that are overexpressed in asthma and chronic obstructive pulmonary disease, and also inhibits antigen presentation [36]. In our study, we found that the IL-10 concentration in serum and the IL-10 mRNA expression in lung tissue homogenate of asthmatic rats were both markedly lower than that of normal rats (*P* < 0.01, *P* < 0.05). These results indicated that IL-10 was involved in the pathogenesis and development of asthma; meanwhile, it can suppress the progression of asthma. Furthermore, puerarin or dexamethasone prevention could both increase the contents of IL-10 in serum and the IL-10 mRNA expression in lung tissue homogenate of asthmatic rats.

Recently, other studies described that asthma hemostatic unbalance is of importance for the perpetuation of allergic inflammation [4]. In our study, we detected the PT, APTT, FIB, and TT changes of rats in different groups. Compared with the normal group, the asthma group had significantly higher PT (*P* < 0.01), FIB (*P* < 0.01), and TT (*P* < 0.05); the APTT was decreased without statistical significance; this results indicated that there is a hemostatic unbalance in OVA-induced asthmatic rats. In order to investigate the target sites of this unbalance in clotting cascade pathway, we tested the activity of related coagulation factors. Compared with the normal group, the asthmatic group presented with higher FXII activity and decreased FII, FV, and FVIII activities (all *P* < 0.05); other factors were also lower in activity without statistical significance. Because of the lower activities of FII, FV, FVII, and FX, we could explain the prolonged effect of PT and TT. Meanwhile, the shortened effect of APTT is due to the significantly higher activity of FXII along with insignificantly lower activity of three other intrinsic coagulation factors (FVIII, FIX, and FXI). Moreover, fibrinogen (FIB) is a kind of acute phase reaction protein; airway inflammation could stimulate fibrocystic hyperplasia and produce more FIB, which further impair the asthmatic hemostatic unbalance.

Compared with the asthma group, the puerarin group showed significantly lower PT (*P* < 0.01), FIB (*P* < 0.01), and TT (*P* < 0.05) levels, and the APTT was also comparatively higher but insignificantly. Importantly, there was no difference between the puerarin group and the normal group. These results indicated that puerarin could correct the hemostatic disorder of asthma. At the same time, in order to know the target sites of puerarin in clotting cascade pathway, we tested the effect of puerarin on coagulation factors. As shown in Figures 5 and 6, test results indicated that puerarin could effectively enhance the activities of FII, FV, FVII, and FX and reduce the activities of FVIII, FIX, and FXII; these data elucidated the shortening of PT (*P* < 0.01) and TT (*P* < 0.05) and the prolonging of APTT, compared with asthmatic group. In addition, puerarin showed many physiological activities, including the improvement of blood circulation [27] and anti-inflammatory activity [37], both of which could eventually lead to the decrease in the levels of FIB.

As shown in Figure 4, dexamethasone treatment could decrease FIB (*P* < 0.05), TT (*P* < 0.01), and PT (*P* > 0.05) and prolong APTT levels, compared with the asthmatic group. Dexamethasone could partly correct the disturbed hemostatic balance in asthma. In dexamethasone-treated rats, the higher activities of FII, FVII, and FX and lower activity of FVIII could rectify the changes of levels of PT, TT, and APTT. The reduction of FIB was due to the anti-inflammatory activity of dexamethasone [38, 39]. Dexamethasone is not a kind of hemostasis drug but can play certain role in hemostasis [40, 41]. However, low dose of dexamethasone (<1.0 mg/kg) could inhibit thrombosis; while high dose of dexamethasone (>1.0 mg/kg) could not inhibit thrombosis, it would decrease the activity of t-PA and increase the level of PAI-1 [42]. The result of our study is in accordance with the latter. According to the results of puerarin and dexamethasone combination group, there is no synergism or antagonism between puerarin and dexamethasone on the prevention of asthma.

Protease-activated receptors (PARs) belong to a family of G-protein-coupled receptors that can be activated by serine proteases via proteolytic cleavage. Increased expression of PAR2 is reported in bronchial epithelium of patients with asthma. All PARs have been detected in lungs, in particular in epithelium and airway smooth muscle. PAR1 and PAR2 have also been found on endothelium, macrophages, and migratory cells, such as mast cells and neutrophils [43]. Thus, PAR2 has been implicated more directly in the pathophysiology of asthma. Meanwhile, PAR2 can be activated by coagulation factors FVIIa, thereby providing a direct link between coagulation and inflammation. In our study, we found that FVII mRNA expression was notably increased in asthmatic rats compared with that of normal rats. Puerarin could significantly downregulate the expression of FVII mRNA in asthmatic rats (*P* < 0.01). At the same time, dexamethasone had a similar but less effective preventive function on asthmatic rats (*P* < 0.05).

In conclusion, puerarin was an effective therapeutic agent for suppressing airway inflammation in a rat model of asthma. Additionally, its effectiveness was found to be better than dexamethasone in balancing the asthmatic hemostasis condition. In this study we provide *in vivo* data for the development of effective and new therapeutic natural product for asthma treatment. However, further studies are required in order to elucidate its detailed mechanism of action.

## Figures and Tables

**Table 1:** Sense and antisense primers used for reverse transcription-polymerase chain reaction (RT-PCR).

Target mRNA	Primer sequences	
GADPH	Sense	5′-GAAGGTGAAGGTCGGAGTC-3′
	Antisense	5′-GAAGATGGTGATGGGATTTC-3′

IL-4	Sense	5′-ACCTTGCTGTCACCCTGTTC-3′
	Antisense	5′-GTTGTGAGCGTGGACTCATTC-3′

IL-10	Sense	5′- GCTATGTTGCCTGCTCTT-3′
	Antisense	5′- CCAAGTAACCCTTAAAGTCC-3′

IFN-*γ*	Sense	5′-ACTCATTGAAAGCCTAGAAAGTC-3′
	Antisense	5′-TCTTCTTATTGGCACACTCTCTA-3′

FVII	Sense	5′-ACTCACGGACTGCTGCTT-3′
	Antisense	5′- CGCCATCGCTGTAAATAG-3′

GADPH: glyceraldehydes-3-phosphate dehydrogenase; IFN-*γ*: interferon-*γ*; IL: interleukin.

## References

[1] Regal JF (2004). Immunologic effector mechanisms in animal models of occupational asthma. *Journal of Immunotoxicology*.

[2] Wills-Karp M (1999). Immunologic basis of antigen-induced airway hyperresponsiveness. *Annual Review of Immunology*.

[3] Elias JA, Lee CG, Zheng T, Ma B, Homer RJ, Zhu Z (2003). New insights into the pathogenesis of asthma. *Journal of Clinical Investigation*.

[4] De Boer JD, Majoor CJ, Van ’t Veer C, Bel EHD, Van Der Poll T (2012). Asthma and coagulation. *Blood*.

[5] Matthay MA, Clements JA (2004). Coagulation dependent mechanisms and asthma. *Journal of Clinical Investigation*.

[6] Masoli M, Fabian D, Holt S, Beasley R (2004). The global burden of asthma: executive summary of the GINA Dissemination Committee Report. *Allergy*.

[7] Eder W, Ege MJ, Von Mutius E (2006). The asthma epidemic. *The New England Journal of Medicine*.

[8] Asher MI, Montefort S, Björkstén B (2006). Worldwide time trends in the prevalence of symptoms of asthma, allergic rhinoconjunctivitis, and eczema in childhood: ISAAC Phases One and Three repeat multicountry cross-sectional surveys. *The Lancet*.

[9] Asthma (2000). With asthma cases on the rise, a better understanding of the disease on the molecular and systems levels promises more efficacious treatments. *Nature Biotechnology*.

[10] Upur H, Abdureyim S, Amat N, Umar A, Berke B, Moore N (2011). Anti-inflammatory, immunomodulatory, and heme oxygenase-1 inhibitory activities of Ravan Napas, a formulation of Uighur traditional medicine, in a rat model of allergic asthma. *Evidence-Based Complementary and Alternative Medicine*.

[11] Walsh GM (2012). An update on emerging drugs for asthma. *Expert Opinion on Emerging Drugs*.

[12] Sheth A, Reddymasu S, Jackson R (2006). Worsening of asthma with systemic corticosteroids: a case report and review of literature. *Journal of General Internal Medicine*.

[13] Wenzel SE, Covar R (2006). Update in asthma 2005. *American Journal of Respiratory and Critical Care Medicine*.

[14] Guilbert TW, Morgan WJ, Zeiger RS (2006). Long-term inhaled corticosteroids in preschool children at high risk for asthma. *The New England Journal of Medicine*.

[15] Aun MV, Ribeiro MR, Costa Garcia CL, Agondi RC, Kalil J, Giavina-Bianchi P (2009). Esophageal candidiasis-an adverse effect of inhaled corticosteroids therapy. *Journal of Asthma*.

[16] Li XM (2009). Complementary and alternative medicine in pediatric allergic disorders. *Current Opinion in Allergy and Clinical Immunology*.

[17] Zhou J, Wang H, Xiong Y, Li Z, Feng Y, Chen J (2010). Puerarin attenuates glutamate-induced neurofilament axonal transport impairment. *Journal of Ethnopharmacology*.

[18] Tian F, Xu L-H, Zhao W, Tian L-J, Ji X-L (2011). The optimal therapeutic timing and mechanism of puerarin treatment of spinal cord ischemia-reperfusion injury in rats. *Journal of Ethnopharmacology*.

[19] Zhang N-B, Huang Z-G, Cui W-D, Ding B-P (2011). Effects of puerarin on expression of cardiac Smad3 and Smad7 mRNA in spontaneously hypertensive rat. *Journal of Ethnopharmacology*.

[20] Pang W, Lan XM, Wang CB (2012). Effect of puerarin on the release of interleukin-8 in co-culture of human bronchial epithelial cells and neutrophils. *Chinese Journal of Integrative Medicine*.

[21] Liu X-J, Zhao J, Gu X-Y (2010). The effects of genistein and puerarin on the activation of nuclear factor-*κ*B and the production of tumor necrosis factor-*α* in asthma patients. *Pharmazie*.

[22] Choo M-K, Park E-K, Yoon H-K, Kim D-H (2002). Antithrombotic and antiallergic activities of daidzein, a metabolite of puerarin and daidzin produced by human intestinal microflora. *Biological and Pharmaceutical Bulletin*.

[23] Pang W, Lan XM (2011). Effect of puerarin on the release of MCP-1 in co-culture of human bronchial epithelial cells and neutrophils. *Journal of Medical Postgraduates*.

[24] Liu Y, Shao LL, Pang W Induction of adhesion molecule expression in co-culture of human bronchial epithelial cells and neutrophils suppressed by puerarin via down-regulating p38 mitogen-activated protein kinase and nuclear factor *κ*B pathways.

[25] Pan H-P, Yang J-Z, Mo X-L (2005). Protection of puerarin on the cerebral injury in the rats with acute local ischemia. *Zhongguo Zhongyao Zazhi*.

[26] Wang Y-L, Liu W, Jiang P (2005). Myocardial protective effect of puerarin injection in children with severe pneumonia. *Zhongguo Zhong Xi Yi Jie He Za Zhi Zhongguo Zhongxiyi Jiehe Zazhi*.

[27] Yao X-J, Yin J-A, Xia Y-F (2012). Puerarin exerts antipyretic effect on lipopolysaccharide-induced fever in rats involving inhibition of pyrogen production from macrophages. *Journal of Ethnopharmacology*.

[28] Lee M-Y, Seo C-S, Ha H (2010). Protective effects of Ulmus davidiana var. japonica against OVA-induced murine asthma model via upregulation of heme oxygenase-1. *Journal of Ethnopharmacology*.

[29] Bochner BS, Busse WW (2005). Allergy and asthma. *Journal of Allergy and Clinical Immunology*.

[30] Bochner BS, Busse WW (2004). Advances in mechanisms of allergy. *Journal of Allergy and Clinical Immunology*.

[31] Holgate ST, Davies DE, Puddicombe S (2003). Mechanisms of airway epithelial damage: epithelial-mesenchymal interactions in the pathogenesis of asthma. *European Respiratory Journal, Supplement*.

[32] Larche M, Robinson DS, Kay AB (2003). The role of T lymphocytes in the pathogenesis of asthma. *Journal of Allergy and Clinical Immunology*.

[33] Bisset LR, Schmid-Grendelmeier P (2005). Chemokines and their receptors in the pathogenesis of allergic asthma: progress and perspective. *Current Opinion in Pulmonary Medicine*.

[34] Xu CQ, JJ LE, Duan XH (2011). Molecular mechanism of icariin on rat asthmatic model. *Chinese Medical Journal*.

[35] Hawrylowicz CM (2005). Regulatory T cells and IL-10 in allergic inflammation. *Journal of Experimental Medicine*.

[36] Barnes PJ (2008). The cytokine network in asthma and chronic obstructive pulmonary disease. *Journal of Clinical Investigation*.

[37] Jun M, Hong J, Jeong W-S, Ho C-T (2005). Suppression of arachidonic acid metabolism and nitric oxide formation by kudzu isoflavones in murine macrophages. *Molecular Nutrition and Food Research*.

[38] Ferreira FS, Brito SV, Saraiva RA (2010). Topical anti-inflammatory activity of body fat from the lizard Tupinambis merianae. *Journal of Ethnopharmacology*.

[39] Heying R, Wehage E, Schumacher K (2012). Dexamethasone pretreatment provides antiinflammatory and myocardial protection in neonatal arterial switch operation. *Annals of Thoracic Surgery*.

[40] Brotman DJ, Girod JP, Posch A (2006). Effects of short-term glucocorticoids on hemostatic factors in healthy volunteers. *Thrombosis Research*.

[41] Appel IM, van Kessel-Bakvis C, Stigter R, Pieters R (2007). Influence of two different regimens of concomitant treatment with asparaginase and dexamethasone on hemostasis in childhood acute lymphoblastic leukemia. *Leukemia*.

[42] Van Giezen JJJ, Brakkee JGP, Dreteler GH, Bouma BN, Jansen JWCM (1994). Dexamethasone affects platelet aggregation and fibrinolytic activity in rats at different doses which is reflected by their effect on arterial thrombosis. *Blood Coagulation and Fibrinolysis*.

[43] Peters T, Henry PJ (2009). Protease-activated receptors and prostaglandins in inflammatory lung disease. *British Journal of Pharmacology*.

